# Grandmothering and cognitive resources are required for the emergence of menopause and extensive post-reproductive lifespan

**DOI:** 10.1371/journal.pcbi.1005631

**Published:** 2017-07-20

**Authors:** Carla Aimé, Jean-Baptiste André, Michel Raymond

**Affiliations:** 1 Institut des sciences de l’évolution de Montpellier. CNRS UMR 5554 –IRD—Université de Montpellier, Montpellier, France; 2 Institut Jean-Nicod. CNRS UMR 8129—Institut d’Etude de la Cognition—Ecole Normale Supérieure–PSL Research University, Paris, France; University of California Irvine, UNITED STATES

## Abstract

Menopause, the permanent cessation of ovulation, occurs in humans well before the end of the expected lifespan, leading to an extensive post-reproductive period which remains a puzzle for evolutionary biologists. All human populations display this particularity; thus, it is difficult to empirically evaluate the conditions for its emergence. In this study, we used artificial neural networks to model the emergence and evolution of allocation decisions related to reproduction in simulated populations. When allocation decisions were allowed to freely evolve, both menopause and extensive post-reproductive life-span emerged under some ecological conditions. This result allowed us to test various hypotheses about the required conditions for the emergence of menopause and extensive post-reproductive life-span. Our findings did not support the Maternal Hypothesis (menopause has evolved to avoid the risk of dying in childbirth, which is higher in older women). In contrast, results supported a shared prediction from the Grandmother Hypothesis and the Embodied Capital Model. Indeed, we found that extensive post-reproductive lifespan allows resource reallocation to increase fertility of the children and survival of the grandchildren. Furthermore, neural capital development and the skill intensiveness of the foraging niche, rather than strength, played a major role in shaping the age profile of somatic and cognitive senescence in our simulated populations. This result supports the Embodied Capital Model rather than the Grand-Mother Hypothesis. Finally, in simulated populations where menopause had already evolved, we found that reduced post-reproductive lifespan lead to reduced children’s fertility and grandchildren’s survival. The results are discussed in the context of the evolutionary emergence of menopause and extensive post-reproductive life-span.

## Author summary

In all human populations, regardless of environmental and socioeconomic conditions, menopause occurs in women well before the end of their expected lifespan. Conversely, extensive post-reproductive life-span is rare in other species; except in some cetaceans. Evolutionary theory predicts that menopause and extensive post-reproductive lifespan should emerge and persist in populations only if it is advantageous for gene transmission. Identifying this advantage is a long-standing issue. We provide a better understanding by demonstrating that humans’ cognitive abilities, in association with grand-mothering, are required for the emergence of this pattern. Indeed, cognitive abilities allow accumulation of skills and experience over the lifespan, thus providing an advantage for resource acquisition. These surplus resources can then be used to increase the number of offspring or be transmitted to existing offspring and grandoffspring. Stopping reproduction during aging allows allocating more resources to assist offspring and grandoffspring, thus increasing children’s fertility and grandchildren’s survival.

## Introduction

Menopause, the permanent cessation of ovulation, occurs in women well before the end of their expected lifespan; reproductive senescence occurs substantially earlier than somatic senescence, leading to a particularly long post-reproductive life [1]. This is a rather uniform pattern across traditional and modern human societies. For example, if a man or a woman reaches age 45, he or she can expect to live at least an additional two decades [2–5]. However, and remarkably consistently across populations, reproductive senescence in women is largely completed by age 45 [6]. Extensive post-reproductive life-span (PRLS) in humans is thus not a consequence of modern improvements to nutrition, hygiene or medicine. Rather, reproductive cessation occurring approximately twenty years before the end of the expected lifespan appears as a constant feature of human biology [5, 7]. Among other species, only pilot and killer whales also exhibit extensive female PRLS. For instance, female killer whales can live into the 90s although they usually stop reproducing around age 40 [8–10]. However, patterns of reproductive and somatic senescence in killer whales differ from those of humans in some other ways, especially for males. Indeed, males rarely live beyond 50 years. Moreover, they do not undergo reproductive cessation [11]. In contrast, observations in traditional human populations have suggested that men may often undergo reproductive cessation once their wives reach menopause [12].

Understanding the conditions involved in the evolution of menopause and extensive PRLS is a long-standing challenge for biologists. First, an early end to reproduction seems contrary to maximizing Darwinian fitness. Second, the selective advantage associated with long life after the end of reproduction is not trivial. Various hypotheses have been proposed (for a review see [13]), including the Maternal hypothesis (MH), the Grand-mother Hypothesis (GMH), and the Embodied capital model (ECM). The MH is the idea that menopause has evolved in humans to avoid the risk of dying at childbirth, which is higher in older women, and to ensure the survival of the last offspring [14,15]. This hypothesis might thus explain why ageing women stop reproduction. However, as it relies on costs but not on benefits, the MH seems unlikely to explain alone the particularly long duration of PRLS observed in women. Indeed, whereas age-related costs of reproduction may explain early end of reproduction, it cannot explain why additional life after reproduction may be advantageous. Furthermore, death in childbirth may not be common enough to constitute a sufficient cost [16]. According to GMH [7] and ECM [12], both menopause and long life after reproduction may have evolved as two parts of the same allocation strategy consisting of ceasing to allocate resources to direct reproduction (*i*.*e*. producing new children) to favor indirect reproduction (*i*.*e* parental or grandparental care). Indeed, menopause and extensive PRLS may allow additional resource allocation to grandoffspring care and, therefore, increased fertility of the children and survival of the grandchildren. There are two main differences between the GMH and the ECM. The first resides in the specific causal hypotheses involved [17]. Indeed, according to the GMH, strength (*e*.*g*. proxied by body size) is the primary determinant of resource production [18–21]. Children productivity is low because foraging requires strength. As human growth is particularly slow, benefit of grand-mothering for grand-children survival and fertility is high, generating selection for older women to increase longevity [21]. According to the ECM, neural capital development and the skill intensiveness of the human foraging niche play the major role in shaping the age profile of resource production and transfers. In traditional societies, a peak of resource production is reached approximately twenty years after the peak of strength (mid-twenties) [12]. This is because earlier-life investments in neural capital lead to later-life energetic returns from such investments, with the consequences that individuals still acquire more resources than they need for survival until age 70 [12]. These extra resources could be used either for direct reproduction or for indirect reproduction. However, if the cost of reproduction increases with age (for instance, due to physiological constraints), it may be more advantageous to use these resources for increasing condition and fertility of the children and grandchildren, rather than increasing the number of children. The second difference between GMH and ECM resides in the fact the ECM is a two-sex model, whereas males may not be considered in the GMH. Indeed, as the traditional hunter-gatherer pattern of production, reproduction, and parental investment depends fundamentally on a cooperative division of labor between men and women, the ECM predicts that both aging women and men may stop producing new children to allocate resources to existing children and grand-children.

To test the MH [22, 23] and the GMH [24, 25], empirical studies have compared the fitness of children and grandchildren of women who experienced different durations of post-reproductive life-span. However, it is unclear if these studies help to understand the emergence or maintenance of menopause and extensive PRLS [26]. Indeed, the conditions favoring their maintenance are not the same as the conditions favoring their emergence. This is because female reproductive strategies in a population alter the social environment and determine the benefits of a trait. This change affects competition for reproductive resources and the average relatedness between interacting individuals [26]. Thus, the evolution of menopause and PRLS should not be studied outside of its ecological context or without considering the feedback between the evolution of this trait and the resulting ecology. To empirically study the evolutionary emergence of extensive PRLS, the fitness of rare mutant females who experience menopause should be compared to the fitness of resident females who do not. However, this is a possibility neither in humans, as menopause and extensive PRLS is already present in all populations [26], nor in our closest relative species, as reproductive senescence in midlife seems to be absent in non-human primates [27]. Regarding the ECM, the prediction that both aging women and men may stop producing new children to allocate resources to existing children and grand-children has been already supported by observations in traditional human populations [12]. However, the relation between neural capital development and skill intensiveness of the foraging niche on the one hand, and the duration of PRLS on the other hand, have not been demonstrated yet.

Here, we tested the MH, GMH and ECM for both the emergence and the persistence of menopause and extensive PRLS using a modeling approach based on life-history theory. Life history theory is the idea that living organisms must divide the total energetic potential available to them over their lifetime to perform different tasks, mainly survival, growth, direct reproduction, and parental care [28, 29]. As this energetic potential is limited, trade-offs occur among these tasks, resulting in different life-history strategies. The first trade-off occurs between immediate and future reproduction (*via* investment in growth and survival). The second trade-off occurs between the quantity and quality of offspring (*i*.*e*., having more offspring *versus* a larger investment in each of them). Modeling the evolution of allocation strategies should allow investigating the conditions for a switch from allocation to direct reproduction to allocation to indirect reproduction, *i*.*e*. for the emergence and persistence of both menopause and extensive PRLS. However, it requires a comprehensive model that considers both all of the allocation decisions that an individual has to make during his or her life, and how these decisions are shaped by complex interactions between genes, environment, and the internal state of the individual at the time when he has to make the decision.

We used Artificial Neural Networks (ANNs [30]) to simulate the evolution of resource allocation strategies, including all types of complex, even unforeseen, trade-offs in populations subject to diverse ecological conditions. Allocation decisions were allowed to freely evolve, and menopause and extensive PRLS emerged under some ecological conditions. We then tested for the following predictions: (1) Under the MH, menopause (and thus extensive PRLS) should not be observed without including age-dependent risk of dying at childbirth in the model; (2) Under both the GMH and the ECM, menopause and extensive PRLS should not emerge, whatever the ecological conditions, if resource transfers to grand-offspring are not allowed; (3) Under the ECM only, cognitive resources, because of delayed benefits of investment in neural development, should be a required condition for the emergence of menopause and extensive PRLS. Note that the ECM, as mentioned before, also predicts that both aging women and men may stop producing new children to allocate resources to existing children and grand-children, a prediction which has been supported by observations in traditional human populations [12]. Due to methodological issues (see limitation section), we did not test this prediction here. We rather focused on the relation between cognitive resources and extensive PRLS, which has never been tested before. Finally, we also tested whether MH, GMH and ECM may explain the persistence of extensive PRLS in simulated populations where this trait has already evolved. In these populations, GMH predicts that mother death at the age of menopause or delayed menopause of the mother should lead to decreased fertility of the children and/or decreased grandchildren survival. MH predicts that, under the same conditions, survival of the children should be decreased.

## Results

### 1. Multiple and complex interactions among the ecological parameters shape the duration of PRLS in simulated populations

**Fig 1:**
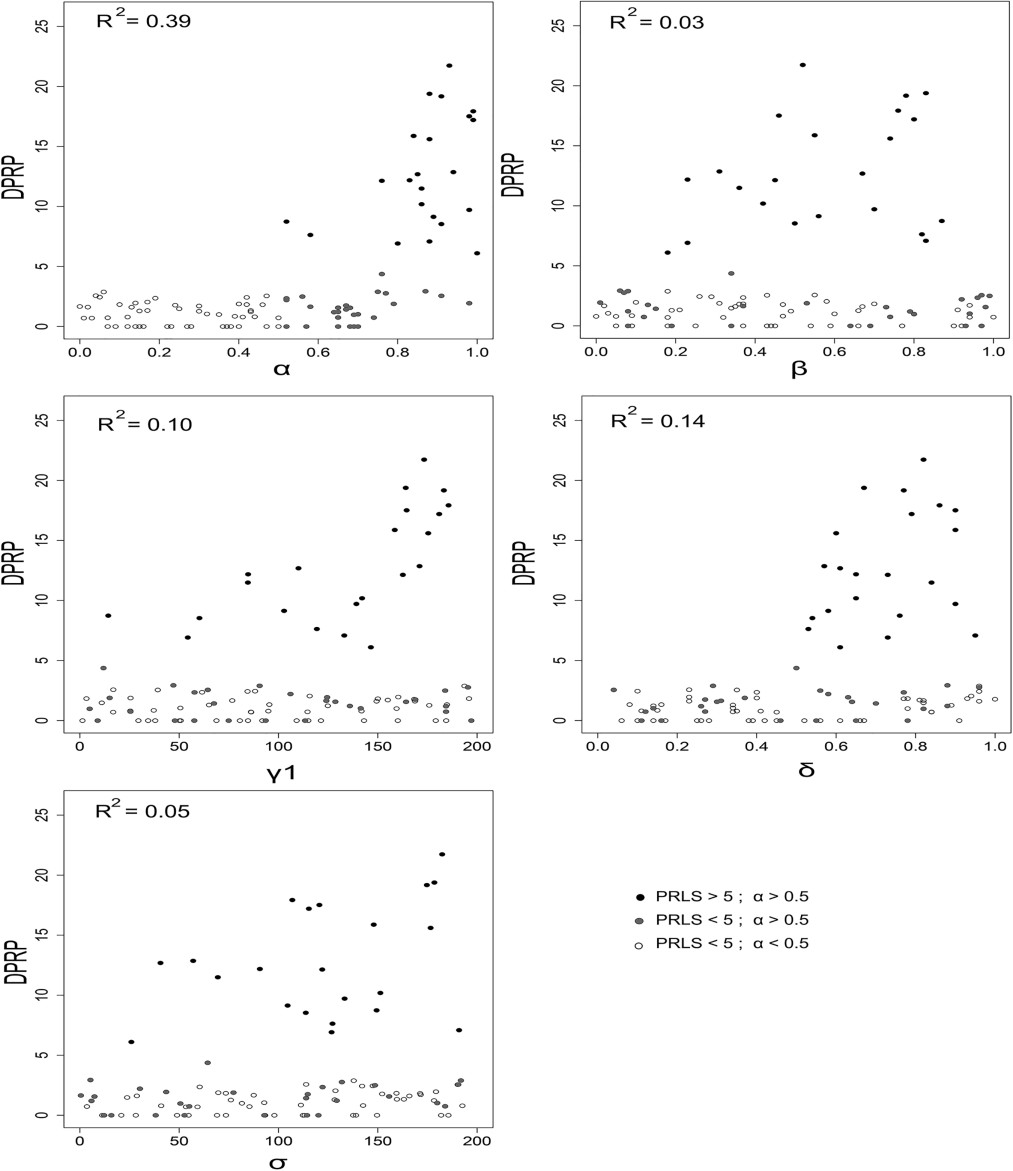
Correlations between the ecological parameters and duration of PRLS. For each observation, a random value was attributed to each of the five ecological parameters (*α*, *β* and *δ* were drawn from a uniform distribution between 0 and 1 and *γ2* and *σ2* from a uniform distribution between 0 and 200), and a simulated population with an initial size of 1,000 individuals was allowed to evolve during 10,000 time units. The duration of PRLS was then measured as the average time interval between the last reproductive event and death, calculated over the individuals who were born and died during the final 2,000 units of time. This process was repeated 100 times to detect the influence of each parameter on the variation of the duration of PRLS.

With the exception of the flow rate of available resources, all of the ecological parameters included in the model (*α*, the skill intensiveness of the foraging niche; *β*, the rate of skills acquisition; *γ2*, the difficulty of acquiring resources in the environment; *δ*, the depletion rate of somatic and cognitive capital; and *σ2*, the dangerousness of the environment) somehow influenced the duration of PRLS (**Fig 1**). Lower values for α were associated with shorter PRLS regardless of the values of the other parameters. However, high values of *α* were not always sufficient to generate a duration of PRLS higher than 5 time units, suggesting the presence of interactions with other parameters. The highest durations of PRLS observed (>5 time units) were associated with parameters of intermediate (for *β*) or high values (for *δ* and *γ2*), suggesting multiple complex interactions among them.

### 2. Evolution of life history traits

**Fig 2:**
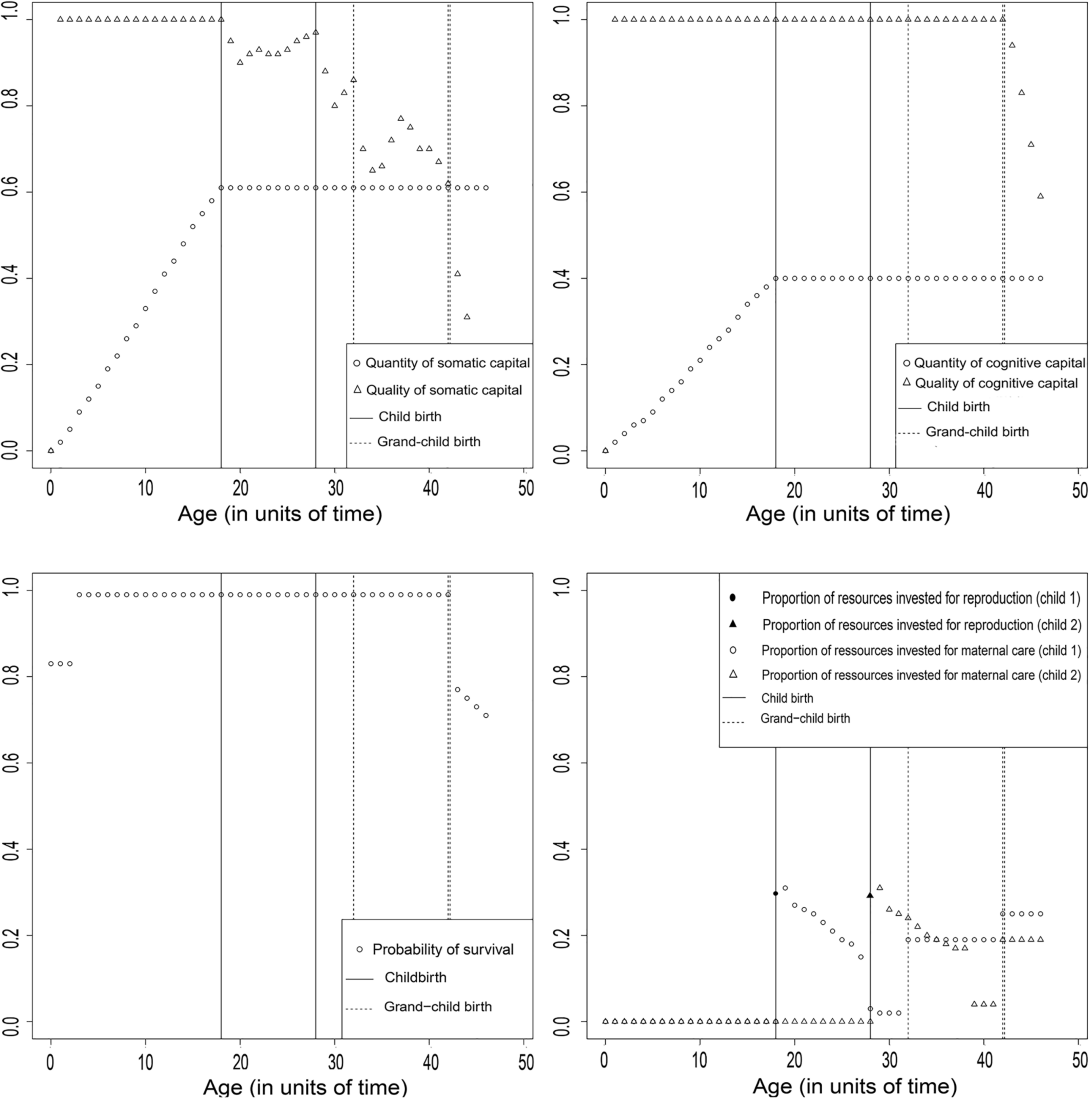
Description of the evolved strategy with the full model and the EP*.

The maximization procedure confirmed that at least one combination of ecological parameters (*α =* 0.91; *β =* 0.47; *γ*_*2*_ *=* 157; *δ = 0*.*87; σ*_*2*_ *= 27)* lead to menopause and extensive PRLS with our model. Among all tested combinations of parameters (see Material and method section), this set, referred to as EP*, led to evolution of the longest duration of PRLS in the simulated population. With EP*, 1,121 individuals were born and died during the final 2,000 time units of the simulation process. Their average duration of PRLS was 21.92 (+/- 3.04) time units. A total of 78.1% of these individuals had exactly the same allocation strategy **(S1 Fig**), as defined by the combination of synaptic weights of the artificial neural network (see the Material and Methods section). Their average duration of PRLS was 18.84 (+/- 0.60). The typical life history of an individual with this allocation strategy achieving reproduction was the following (**Fig 2)**: The first resource allocation was to growth, survival and maintenance until the quantity of somatic capital reached the value of 0.6 (at *t* = 18). Then, the first reproductive event occurred and somatic senescence started, thus suggesting that resources were allocated to reproduction at the expense of investment in the quality of somatic capital. Investment in maternal care for a given child was maximal following birth and then decreased over time. A second reproductive event occurred at *t* = 28, again at the expense of the quality of somatic capital. At *t* = 32, a grandchild was born. Then, the individual started to allocate resources for maternal care for both the first child, who has given birth, and the second child, who is not autonomous yet. The resulting increase in maternal care occurred at the expense of the quality of somatic capital but also at the expense of investment in direct reproduction. Indeed, no individual along the simulation process gave birth to an additional child after the birth of a grandchild. Despite the decrease of the quality of somatic capital, the quality of both cognitive capital and survival probability remained stable until *t* = 42. Then, they started to decrease until death, which occurred at *t* = 47.

### 3. Grand-mothering and cognitive resources are required for the emergence of menopause and extensive PRLS

Whatever the ecological parameters used, menopause and extensive PRLS did not emerge in the simulated populations when grandoffspring care was not allowed. In that case, the mean duration of PRLS obtained after applying the maximization procedure was 1.6 time units, a 92.6% reduction compared to the mean duration of PRLS (21.9 time units) obtained when grand-mothering was a possible option. Similarly, menopause and extensive PRLS did not emerge when cognitive resources were not differentiated from somatic resources in the model (*i*.*e*. both resources are interchangeable and had the same properties, including no delayed benefits). In that case, the mean duration of PRLS obtained after applying the maximization procedure was 2.1 time units, a 90.6% reduction compared to the mean duration of PRLS obtained with the full model. Finally, allowing resource transfers between siblings lead to no substantial changes in the results (duration of the PRLS of 22.04 with the full model, 1.71 without grand-mothering, and 2.03 without delayed benefit of investment in cognition).

### 4. Cost analysis

**Fig 3:**
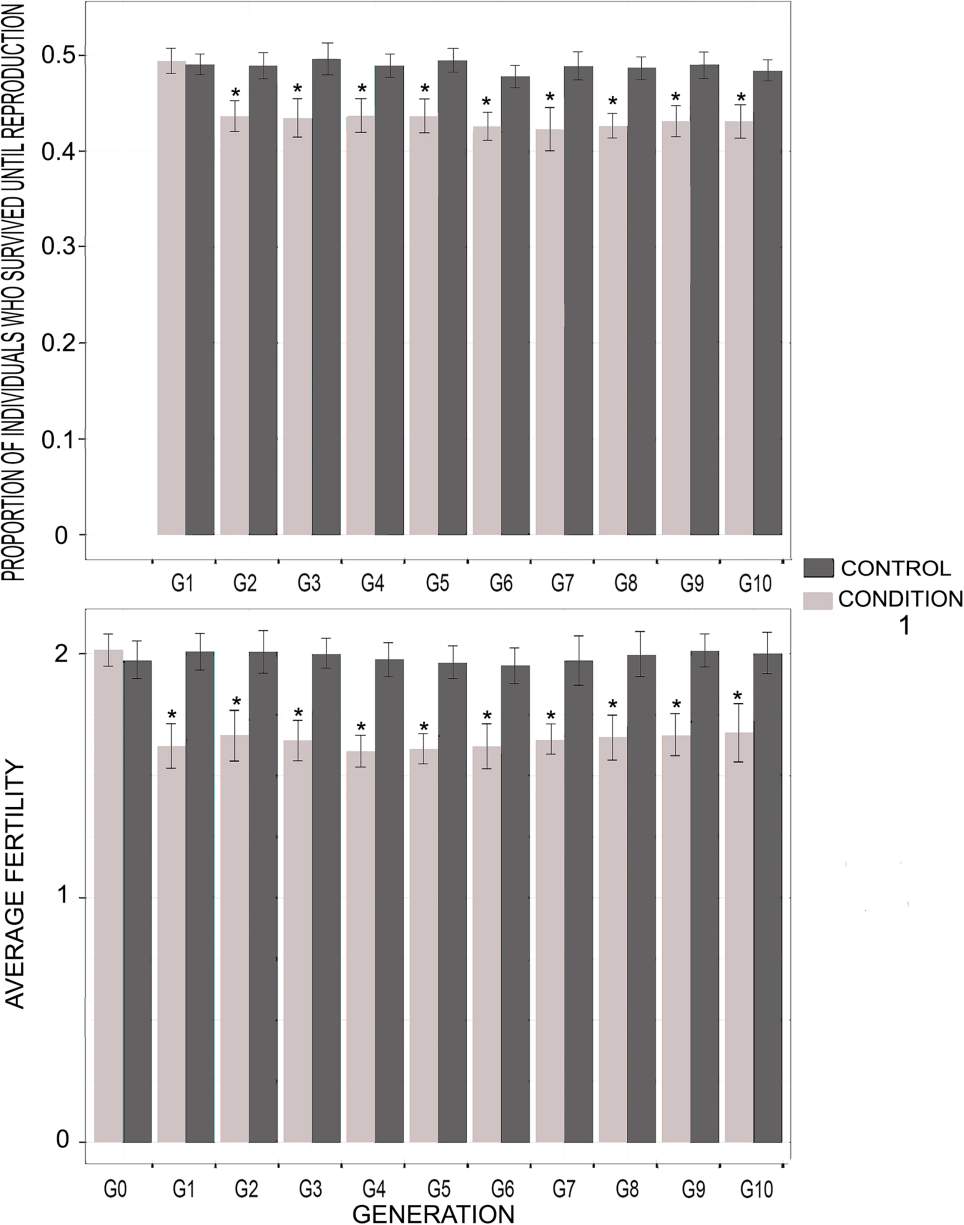
Probability to survive until reproduction and fertility of the individuals receiving condition 1 *versus* control. *: p-value < 0.05. G0 to G10: generation 1 to 10. We simulated 100 populations using the EP* and best synaptic weights, without allowing mutation. All the individuals thus had the same allocation strategy. At *t* = 10,000, we applied condition 1 (death at time of menopause) to half of the individuals in each population, and then to their offspring at each generation. No condition was applied to the other individuals (control). This figure represents the proportion of individuals who survive until reproduction and the average fertility among the individuals receiving the condition, compared to control. Results were averaged and the standard error was calculated over the 100 populations. Significance was assessed using two-sided student tests (R-based function t-test).

**Fig 4:**
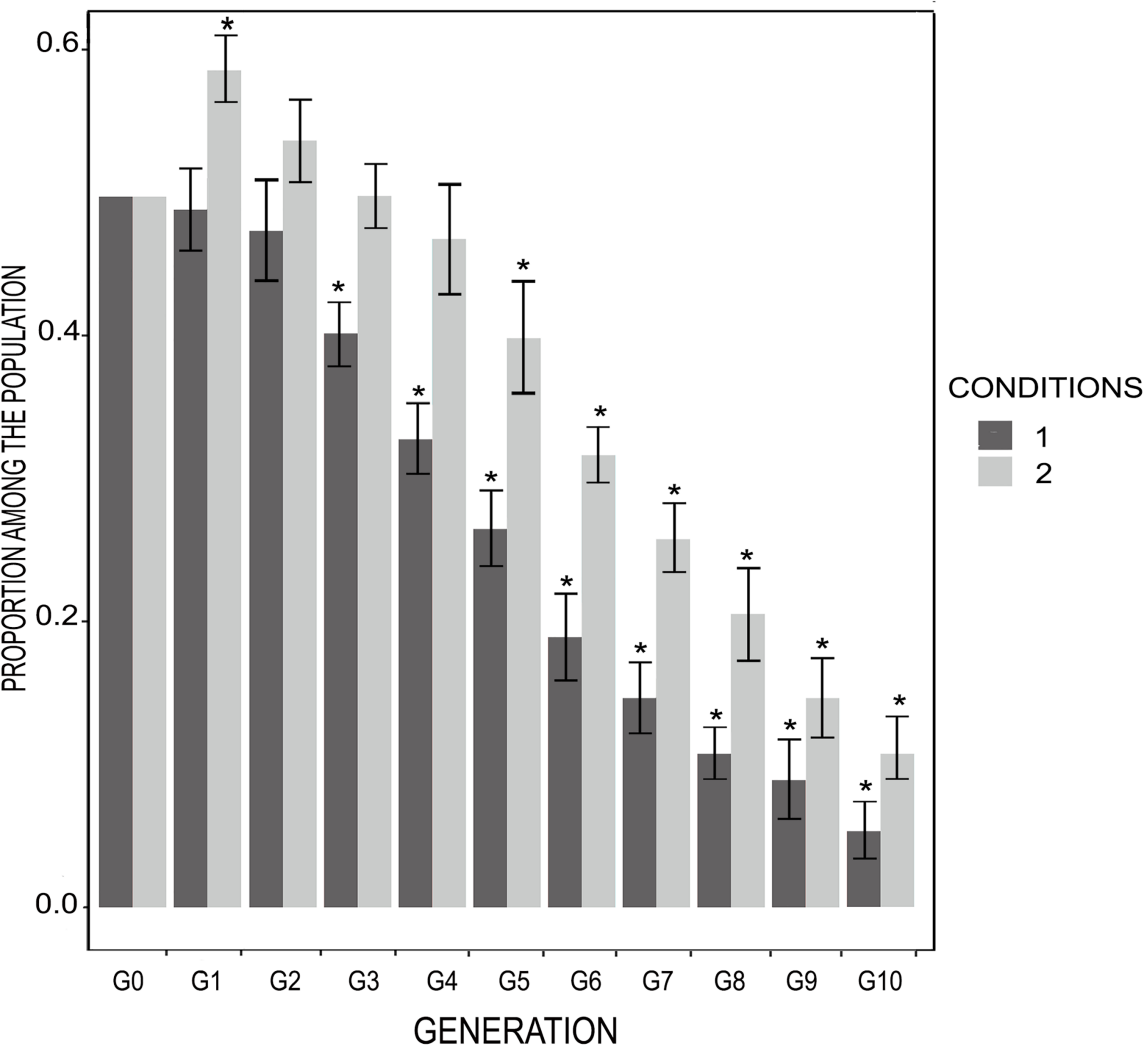
Proportion of individuals receiving condition 1 or 2, from generation G0 to G10. *: p-value < 0.05. We simulated 200 populations using the EP* and best synaptic weights, without allowing mutation. 100 populations were attributed to condition 1 (death at the age of menopause), and 100 populations were attributed to condition 2 (one additional reproductive event at the age of menopause). At *t* = 10,000, we applied condition 1 or 2 to half of the individuals in each population. The condition was heritable and was also applied to their offspring at each generation. No condition was applied to the other individuals and their offspring (control). This figure represents the proportion of individuals who received the condition among the successive generations (up to the 10^th^). Proportions were averaged over populations attributed to condition 1 (black bars), and condition 2 (grey bars). Departure from the expected frequency (0.5) was tested for each condition and generation using two-sided binomial tests (R-based function binom.test), and demonstrate that the condition represents a cost for fitness.

**Fig 5:**
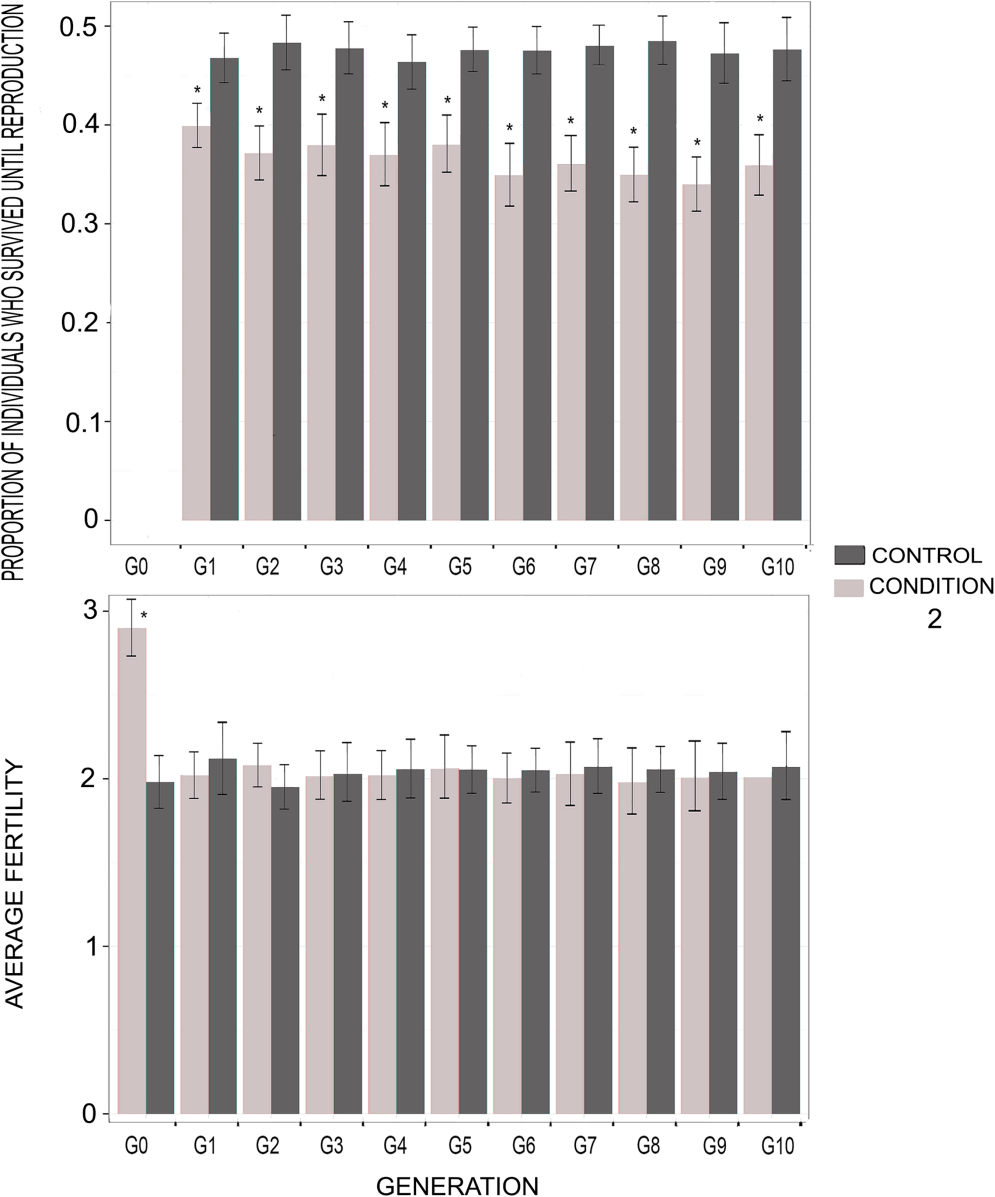
Probability to survive until reproduction and fertility of the individuals receiving condition 2 *versus* control. *: p-value < 0.05. G0 to G10: generation 1 to 10. We simulated 100 populations using the EP* and best synaptic weights, without allowing mutation. All the individuals thus had the same allocation strategy. At *t* = 10,000, we applied condition 2 (one additional reproductive event at time of menopause) to half of the individuals in each population, and then to their offspring at each generation. No condition was applied to the other individuals (control). This figure represents the proportion of individuals who survive until reproduction and the average fertility among the individuals receiving the condition, compared to control. Results were averaged and the standard error was calculated over the 100 populations. Significance was assessed using two-sided student tests (R-based function t-test).

In populations where menopause and extensive PRLS had already evolved, condition 1 (death at the age of menopause) had no detectable effect on the survival of the first-generation children (G1), although these children had reduced fertility. In the subsequent generations, the survival and fertility of the manipulated individuals with condition 1 were lower than the control (**Fig 3**) and they decreased in frequency (**Fig 4**). Manipulated individuals with condition 2 (delayed menopause, *i*.*e*. one additional reproductive event at the age of menopause) were significantly more frequent in the population than control individuals at *G1*, which was expected given the nature of the condition. Then, they decreased in frequency and were significantly less frequent than the control individuals from *G5* to *G10* (**Fig 4**). The probability to survive until reproduction was significantly lower for the manipulated individuals with condition 2 than for the control individuals, from *G1* to *G10*. At *G1*, this difference was explained by a low probability of survival (0.23) for the last child, who was born at the mother’s expected age of menopause. Conversely, the other children had a probability of surviving until reproduction of 0.48, which is equal to those of the control individuals. The fertility of the manipulated individuals with condition 2 was significantly higher than that of the control individuals at *G0*, as expected given the nature of the condition. Then, however, fertility of the manipulated individuals was not significantly different from that of the control individuals from G1 to G10 (**Fig 5**). Finally, the lifespan of the manipulated individuals with condition 2 was significantly shorter than that of control individuals (on average 42 units of time rather than 47, p-value: 0.003).

## Discussion

### 1. Multiple and complex interactions among ecological parameters shape the duration of PRLS in simulated populations

Studying the correlations between the ecological parameters used for the simulations and the resulting duration of PRLS revealed that high values of α (*i*.*e*. skill intensiveness of the ecological niche) were necessary to generate duration of PRLS higher than 5 time units. This result supports the ECM [12]. However, high values of α were not sufficient to generate a duration of PRLS higher than 5 time units, suggesting the presence of complex interactions with other parameters. Therefore, studying the correlations between ecological parameters and the resulting duration of PRLS was insufficient to clearly understand the conditions favoring the emergence of menopause and extensive PRLS. We thus used the maximization procedure to investigate the evolution of life history traits in the simulated populations and to identify required conditions for emergence of menopause and extensive PRLS.

### 2. Evolution of life history traits

When allocation decisions were allowed to freely evolve in a simulated population, menopause and extensive PRLS emerged under at least one set of ecological parameters (**Fig 2**). The patterns of somatic and reproductive senescence obtained were strikingly similar in some ways to those observed in traditional human populations [12]. In particular, we observed a cognitive senescence beginning about twenty units of time after somatic senescence, and stable productivity until cognitive senescence begins. In contrast, some other characteristics of the evolved strategy were less realistic when compared to observations in traditional human populations (*e*.*g*. number of offspring per individual, inter-birth intervals, see **Fig 2**). However, note that we did not aim here to simulate precisely all the aspects of a human life-cycle. Indeed, there are substantial differences in the timing of life-history between human populations around the world, and all this variability cannot be captured here. Moreover, there is no indication that the trait values observed now in hunter-gatherers (mainly living in marginal habitats), reflect the values in the ancestral hunter-gatherers, at a time when menopause evolved. For these reasons, we have designed the maximization procedure to optimize the ecological parameters in order to obtain the longest PRLS under various simulated conditions. This approach allowed us to identify some factors which are required for the emergence of extensive PRLS, whatever the ecological parameters used, the species considered, and the other characteristics of the allocation strategy. An advantage from this approach is that our findings apply to any species with menopause and extensive PRLS, not only humans. Another is that we assumed a minimal number of physiological or environmental constraints.

In particular, menopause and extensive PRLS evolved without imposing a starting condition with the presence of a somatic senescence. Rather, somatic senescence, reproductive senescence (*i*.*e*. menopause) and extensive PRLS evolved as an allocation strategy. Similarly, no prior assumption was made on an increase of the cost of direct reproduction with age. To explain reproductive senescence, MH assumes that the cost of direct reproduction increases with age due to the higher risk of dying at childbirth [14, 15]. ECM also assumes increasing costs of reproduction due to physiological constraints (*e*.*g*. decreasing oocyte quality), although Kaplan *et al*. [12] recognized that additional costs to late-life reproduction beyond physiological costs (*e*.*g*. reduced future productivity from maternal depletion) may exist. Here, the cost of direct reproduction is only defined by the amount of resources allocated for direct reproduction and for parental care, which are allowed to freely evolve. However, when individuals were forced to reproduce at the age of menopause, their own lifespan was significantly reduced, and the child had a higher probability of dying before achieving reproduction, compared to previous children. Late reproduction is thus costly for survival and weakly advantageous for gene transmission, as assumed by MH and ECM. However, this is a result of an evolved allocation strategy rather than the consequence of pre-existing physiological constraints. Similarly, mortality was only a probabilistic consequence of a reduced quantity of resources invested in survival. Extrinsic mortality was not included in the model, as it can be considered that evolved organisms exert some control over many possible causes of mortality (*e*.*g*., by altering patterns of travel to avoid predators, by investing in immune functions, etc.; see [31]). Most types of mortality could thus be seen as the result of an allocation strategy.

### 3. Grand-mothering and skill intensiveness of the ecological niche are required for the emergence of menopause and extensive PRLS

Investigating how patterns of reproductive senescence were shaped by the evolution of allocation decisions under different simulated conditions allowed us to test three hypotheses (MH, GMH and ECM) for the emergence and the persistence of menopause and extensive PRLS. By supporting key assumptions from the GMH and ECM (but not the MH), our results support the idea that both grand-mothering (GMH) and cognitive resources (ECM) are required for the emergence of menopause and extensive PRLS.

#### Maternal hypothesis

The MH assumes that the risk of maternal mortality (defined as mortality during childbirth) associated with late reproduction represents a cost (for the survival of existing children, which would be deprived of maternal investment) that is higher than stopping direct reproduction, contributing to the emergence of menopause [14,15]. Here, MH thus predicts that menopause should not evolve without assuming that the risk of dying at childbirth increases with age. However, at least one set of ecological parameters led to menopause in our simulations in the absence of this assumption. Thus, according to our results, the MH is not a pivotal factor for menopause to emerge, as the risk of dying with a new childbirth was not a selective force to stop reproducing. Maternal mortality during childbirth is minimal in non-human primates [32] and thus probably also in humans during the emergence of menopause. It is therefore possible that the relatively high maternal mortality during childbirth in humans is only a derived condition, occurring during or after the emergence of menopause, rather than a determinant of menopause.

#### Grandmother hypothesis

The GMH assumes that the reproductive costs of stopping direct reproduction are outweighed by grand-mothering, *i*.*e*., the increase of fecundity of the children and survival of the grand-children by specific resource allocation. This hypothesis has been supported by a comparative analysis of birth intervals in mammals; the birth interval in humans is shorter than expected for their size, a possible result of the effect of grandmothers on the fertility of their daughters [7]. In our model, grand-parenting was no longer a resource allocation possibility when investment in a child who already reproduced was set to 0 (see material and method section). The GMH thus predicts that menopause and extensive PRLS would not emerge under these conditions whatever the ecological parameters used, whereas both should emerge under at least some ecological conditions when grand-mothering is allowed. As the results were consistent with this prediction, this study supports the idea that grand-mothering is a required condition for the emergence of menopause and extensive PRLS.

#### Embodied capital model

Our finding that grand-parenting is a required condition for the emergence of extensive PRLS was expected in the contexts of both the GMH and the ECM. However, ECM uniquely predicts that cognitive resources, because of the delayed benefits of investing in brain performance [12], may also be required. Here, according to the ECM, we expected that menopause and extensive PRLS should not emerge without including these delayed benefits in the model, whatever the model parameters used. Results were consistent with this prediction. In addition to grand-mothering, delayed benefits of investment are thus a required condition for the emergence of extensive PRLS. This finding strongly supports the idea that the skill intensiveness of the ecological niche and the importance of cognitive abilities in determining productivity in humans may have allowed for the emergence of extensive PRLS. Indeed, cognitive resources are characterized by strong delayed benefits of investment, as investing in neural development at time *t* promotes accumulation of skills and experience all along the life [12]. However, the ECM as described by Kaplan *et al*. [12] assumed that the cost of direct reproduction increases over age due to physiological constraints (*e*.*g*., diminishing oocyte quality). In non-human primates, it is not clear if the cost of direct reproduction increases with age. For example, an increase of birth interval with age (*e*.*g*., in chimpanzees [33]) could be explained by a decreasing somatic ability to extract energy from the environment and a constant cost of direct reproduction; in baboons, there are (non-significant) indications that offspring of the highest parity (oldest) mothers may be at a slight disadvantage [34]. It is thus unclear from studies of wild non-human primates if an increase in the cost of direct reproduction with age is an ancestral or a derived condition. Regardless, we went further here by demonstrating that this assumption was not needed here for the emergence of menopause and extensive PRLS. Interestingly, the ECM also predicts that selection favors a minimal decrease of cognitive performance with age, compared to physical condition, and this allocation strategy is clearly present in the simulated populations (**Fig 2**). A very late cognitive senescence has been observed in forager-farmers, in particular for semantic fluency [35], and has been confirmed by a genomic study reporting a genetic signature of past selection for persistent cognitive competence at post-reproductive ages in humans [36].

### 4. Cost analysis

We also support the importance of GMH, but not MH, in explaining the persistence of extensive PRLS in populations where this trait has already evolved. Indeed, in a population where extensive PRLS had already evolved, when maternal mortality was enforced at the age of menopause (*i*.*e*., on average 4.3 time units after the second childbirth), the children’s fertility was affected, but not their survival until reproduction (**Fig 3**). This non-reduced survival of motherless children did not result from allocare [37], as children without their mother could not receive resources from other individuals. Rather, it was the result of an evolved strategy consisting in prioritizing survival rather than fertility when facing a lack of resources. In contrast, grand-mothering is likely pivotal to maintain extensive post-reproductive life-span once it has evolved. Indeed, when the grand-mothering effect was suppressed at the age of menopause (the grandmother was forced to die) or reduced (the grandmother was forced to have an additional child so that parental resources were reduced for any given child), this was associated with a reduced fitness and the corresponding strategy decreased in frequency (**Figs 3 and 5**). This is consistent with several empirical studies [23–25, 28–42].

### 5. Limitations and perspectives

The main limitations in this study were due to the use of a one-sex model. Up to now, no validated and reliable method has been published to use neural networks in the context of a two-sex diploid model. We hope that further methodological developments will allow overcoming this limitation in the near future. It would make possible to complement this study by testing another key prediction of the ECM, *i*.*e*. both aging women and men may stop producing new children to reallocate resources to existing children and grand-children. Note however that this prediction has been already supported by observations in traditional populations [12]. In contrast, the relation between cognitive resources and duration of the PRLS had not been previously tested. Using a two-sex model would have also allowed testing the reproductive conflict hypothesis [23, 39, 43]. The idea is that, when old and young women are co-breeding in the same family unit, as in patrilocal societies, menopause could be the result of a limitation in resources due to competition. Relatedly, some authors suggested that, in this context of intra-familial competition, younger females should benefit from a decisive advantage as compared to older females [25,43,44] due to asymmetric relatedness. Indeed, the daughter-in-law is not related to the children of her mother-in-law, but the mother-in-law is related to the children of her daughter-in-law. Testing of these hypotheses require using a two-sex model, as they are explicitly based on relatedness within a family. Therefore, it cannot be excluded here that these processes, in addition with grand-mothering and cognitive resources, may have also played a role in the emergence or persistence of menopause and extensive PRLS in humans.

More generally, future developments may be envisaged to make the model more realistic. For instance, this may include taking into account migration and patterns of patri or matri-locality (*i*.*e*. the individuals can invest for their kin only if they are co-resident), modelling resource transfers between non-kin or distant kin, considering separately different kind of resources (*e*.*g*. time and energy), or allowing different degradation rates for somatic and cognitive capital. Indeed, in the absence of any published evidence that the respective degradation rates of somatic and cognitive capital are different, for the sake of simplicity, we assumed that these two rates are equal. Note that we speak here of physiological degradation rates, which are different from observed rates of decrease in performance. Indeed, there is published evidence that age-related decline in physical strength follows a very different trajectory than age-related decline in various cognitive abilities [35, 45]. However, age-related decline in performance depends both on the physiological degradation rate and on investment in maintenance. Finally, we assumed here a maximum of five children living simultaneously. This assumption was also imposed by computational limits. However, this is unlikely to have affected the results, as no individuals gave birth to more than three children in our simulated populations using the EP* parameters.

To conclude, we hope to stimulate further interest to use artificial neural networks (or any other adequate tool) to study the evolution of allocation decisions to address these questions, as well as many other issues in evolutionary biology. Indeed, allocation decisions are central to various long-standing questions in this field (*e*.*g*., the evolution of senescence, cognition, social interactions,…), and modelling their evolution may result in significant improvement.

## Materials and methods

### 1. Model description

**Fig 6:**
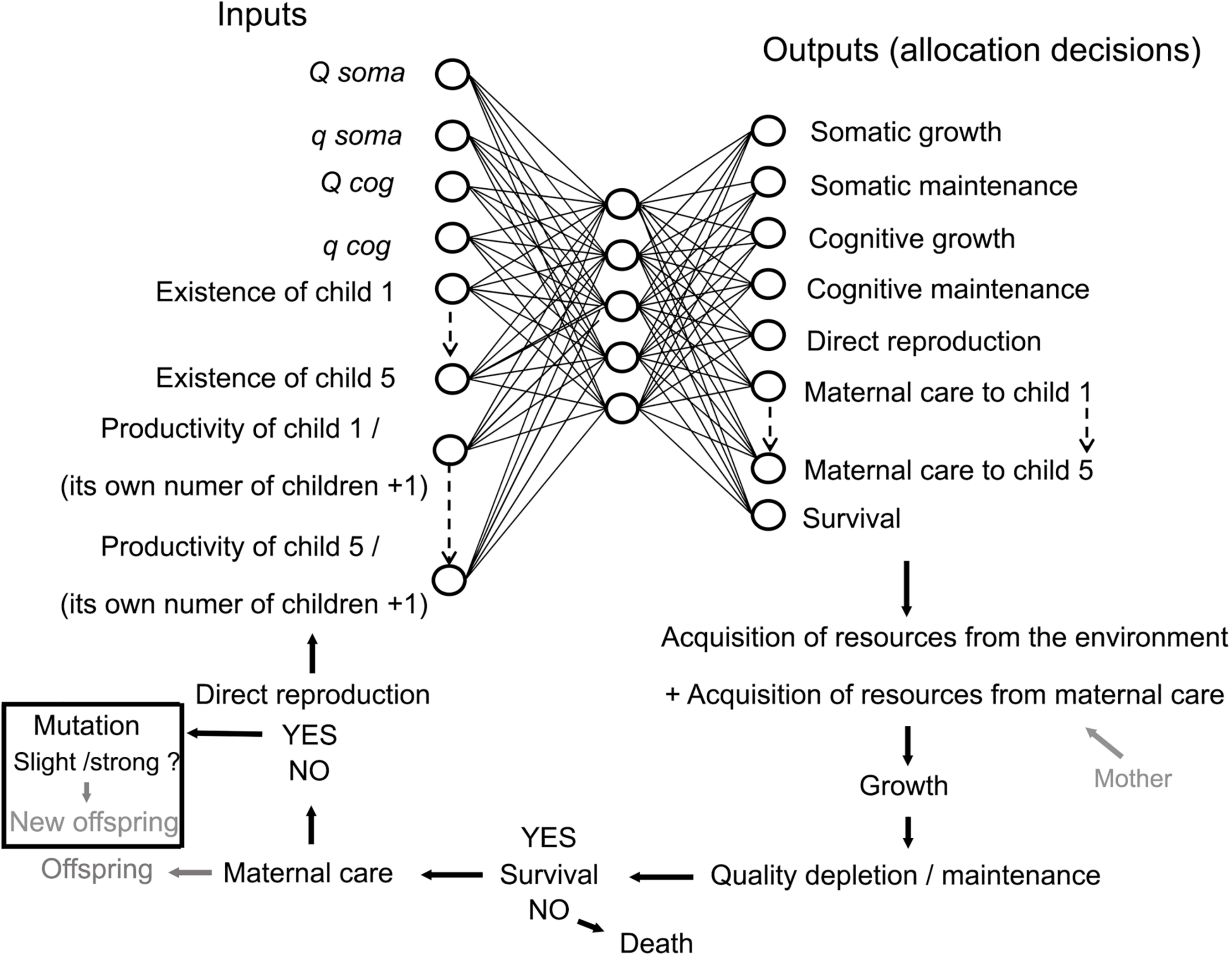
Schematic representation of the model. Qsoma: Quantity of somatic capital. Qsoma: Quality of somatic capital. Qcog: Quantity of cognitive capital. Qcog: Quality of cognitive capital.

The model (**Fig 6**) was coded in C++. The neural networks were fully connected multi-layer perceptrons with a single hidden layer of 5 neurons. The inputs to the networks were information on the internal state and social environment perceived by the individual. The outputs were the proportions of resources allocated to each function.

#### Input neurons

Each individual had 14 input neurons (plus a bias neuron, whose value is always 1). Each of them provided distinct information to the individual about her own current state (inputs 1–4) or social environment (inputs 5–14), as follows:

*Input 1*. *Quantity of somatic capital*: Somatic capital corresponds to all of the organized somatic tissues (muscles, digestive organs, *etc*.) that compose an individual, except those associated with cognitive functions [25]. *Input 2*. *Quality of somatic capital*: Quality is to be understood here as cell quality. As maintaining many cells is costly, the quality of the somatic capital depletes over time proportionally to its quantity. We expressed the quality of somatic capital at time *t* as the proportion (ranging from 0 to 1, with 0 = zero percent and 1 = one hundred percent) of somatic capital which was not degraded. *Input 3*. *Quantity of cognitive capital*: Cognitive capital is composed of the tissues associated with cognitive functions (*i*.*e*., the brain [43]). *Input 4*. *Quality of cognitive capital*: Quality of cognitive capital is a similar concept to the quality of somatic capital, applied to the tissues associated with cognitive functions. *Inputs 5 to 9*. *Children*: The number of children was coded as one binary input per child (0: non-existent/1: existent), with a maximum of five children living simultaneously. *Inputs 10 to 14*. *Productivity of each child*: The children’s productivities were also determined by one input per child. Productivity quantifies the ability of an individual to extract resources in the environment (see the “procedure” section). To consider the existence of grandchildren, inputs corresponding to productivities of each child were corrected by their own number of children (*i*.*e*., divided by the number of children, plus one to avoid divisions by zero).

#### Hidden neurons and synaptic weights

Connections between input and hidden neurons and between hidden and output neurons represent value modifications (activation), via activation functions. Each output neuron is connected to each hidden neuron and its value after activation is the total of the activations received from each hidden neuron. The activation functions were logistical functions using as parameters a set of values called synaptic weights. The combination of synaptic weights of one simulated individual determined its allocation strategy. At the start of each simulated population, the synaptic weights were randomly assigned for each individual from a uniform distribution ranging between -10 and 10.

#### Output neurons

Each individual had 11 output neurons, which represented the proportion of the resources obtained at time *t* which are allocated for each of the following functions: *output 1*: *somatic growth*, *output 2*: *somatic maintenance*, *output 3*: *cognitive improvement*, *output 4*: *cognitive maintenance*, *output 5*: *survival*, *output 6*: *direct reproduction*, and *outputs 7 to 11*: *maternal care for each child*. Outputs were normalized to obtain a total of 1.

#### Initial state

At time *t* = 0, the value of all of the input neurons was 0 (as for new-born individuals) and a pool of 200 initial resources was attributed to each individual.

#### Procedure

At each unit of time, several successive events occurred for each individual (**Fig 6**):

1. *Calculation of the values of the output neurons*: The value of the output neurons was calculated based on the values of the input neurons at time *t* and the set of synaptic weights of the individual.
2. *Acquisition of resources based on productivity*: The flow rate of the resources available for the population is limited, allowing to model competition and to limit population expansion. Indeed, at each time unit, a pool of 20,000 units of resources was available and divided between the individuals depending on their productivity (*i*.*e*., ability to extract resources from the environment). The productivity of each individual is a function of the quantity and quality of both somatic and cognitive capitals. The respective weights of somatic and cognitive capital to determine productivity (*P*) were computed using the Cobb-Douglas function [46]:
  $$P=K^{\alpha -1}.C^{\alpha }$$ (1)
  where K is the somatic factor, C is the cognitive factor, and α is an ecological parameter which defines the relative importance of the somatic and cognitive factors to determine the ability to extract resources from the environment, given the ecological niche. As we defined productivity as a product of the somatic and the cognitive factors, total compensation of one type of resources by the other is not possible (*e*. *g*. a value of 0 for somatic capital cannot be compensated by higher value for cognitive capital). K is defined as:
  $$K=Q_{soma}.q_{soma}$$ (2)
  where *Q*_*soma*_ is the quantity of somatic capital, and *q*_*soma*_ is the quality of the somatic capital. Quality of the somatic capital is thus to be understood as the proportion of somatic capital which is not degraded. C is defined as:
  $$\begin{array}{c} C=Q_{{cognitive}_{t}}.q_{{cognitive}_{t}}.\beta .\int_{t=0}^{t}Q_{cognitive}.q_{cognitive} \end{array}$$ (3)
  where *Q*_*cognitive*_ is the quantity of cognitive capital, *q*_*cognitive*_ is the quality of the cognitive capital, and *β* is the rate of skills acquisition. The integral term in *Eq 3* represents the delayed benefits of investing earlier in cognitive capital. Indeed, investing in brain performance (*i*.*e*., quantity or quality of cognitive capital) at time *t* allows an individual to acquire skills that will be useful in the future to acquire resources. Finally, *P* is transformed using a sigmoid function of the following form:
  $$P^{\prime }=(\frac{1}{1+e^{\gamma 1.(\gamma 2-P)}}-\frac{1}{1+e^{\gamma 1.\gamma 2}}).\frac{1+e^{\gamma 1.\gamma 2}}{e^{\gamma 1.\gamma 2}}$$ (4)
  where γ1 is the slope of the sigmoid function at the inflexion point and γ2 is the abscissa of the inflexion point. We chose a sigmoid function to model the age-dependence of resource acquisition. Indeed, in most mammals, including humans, young individuals are unable to acquire resources by themselves. Then, they become increasingly efficient and finally, saturation occurs. The sigmoid function has been scaled to have *P’* = 0 when *P* = *0* and *P’*→*1* when *P*→+∞.
3. *Acquisition of maternal resources*: At age 0, an individual acquired only the resources which her mother has allocated for direct reproduction. Later, an individual acquired the resources that the mother had eventually allocated for maternal care.
4. *Growth*: A proportion of the newly acquired resources, as determined by outputs *(1)* and *(3*), was allocated to growth. This means that these resources were added to the quantity of somatic and cognitive capital, respectively.
5. *Depletion of the quality of somatic and cognitive capitals and investment for maintenance*: Without investment for maintenance, the quality of somatic and cognitive capital depreciates over time due to environmental assaults and the accumulation of deleterious by-products of cell metabolism [25]. Indeed, at each time, the quality of both somatic and cognitive capital decreases proportionally to their respective quantity. More precisely, the amount of somatic capital which is not degraded (*Q*_*f*_) decreased at each unit of time following a function of the following form:
  $$Q_{f_{t+1}}=Q_{f_{t}}–Q.\delta +I_{m}$$ (5)
  where *δ* is the degradation rate (0 < *δ* <1) and *I*_*m*_ is the amount of resources allocated for maintenance. Then, the quality of capital (somatic or cognitive) is:
  $${q_{t}=Q}_{f_{t}}/Q$$ (6)
  We assumed the same degradation rate for both somatic and cognitive capital.
6. Survival: The probability to survive from time t to time t+1 was a sigmoid function of allocation in survival at time t:
  $$s=(\frac{1}{1+e^{\sigma 1.(\sigma 2-I_{s})}}-\frac{1}{1+e^{\sigma 1.\sigma 2}}).\frac{1+e^{\sigma 1.\sigma 2}}{e^{\sigma 1.\sigma 2}}$$ (7)
  where σ1 is the slope of the sigmoid function at the inflexion point, σ2 is the abscissa of the inflexion point, and *I*_*s*_ is the quantity of resources invested in survival. The function has been scaled to have *s = 0* when *I*_*s*_ *= 0* and *s→1* when *I*_*s*_ *→+∞*. This sigmoid function has been chosen for similar reasons than in Eq (4). An individual who dies at time t cannot, at the same time, allocate resources to other individuals (direct reproduction and parental care).
7. *Reproduction*: If allocation to direct reproduction was higher than zero, the individual reproduced. A new individual was thus added to the population, with the same synaptic weights, except when mutation occurred. For each synaptic weight, the probability of mutation was established as one divided by the total number of weights (1/141). When a mutation occurred, it was either strong (with a probability of 0.1) and the new synaptic weight was drawn from a uniform distribution ranging from -10 to 10, or it was weak (with a probability of 0.9) and the new synaptic weight was drawn from a normal distribution centered on the previous value, with a standard deviation of 0.01.
8. *Maternal care*: The proportion of resources transmitted to each child was determined by one distinct output per child.

### 2. Effects of the ecological parameters on PRLS

Preliminary exploration showed that increasing the amount of available resources at each time unit, all else being equal, lead only to a proportional increase in population size, without changing the average duration of post-reproductive period. This parameter was established at 20,000, resulting in population sizes of at least 500 individuals. A random value was attributed to each of the five other parameters (*α*, *β* and *δ* were drawn from a uniform distribution between 0 and 1 and *γ2* and *σ2* from a uniform distribution between 0 and 200), and a simulated population with an initial size of 1,000 individuals was allowed to evolve during 10,000 time units with these parameters. PRLS was measured as the average time interval between the last reproduction and death, calculated over the individuals who were born and died during the final 2,000 units of time. This process was repeated 100 times to detect the influence of each parameter on the variation of PRLS.

### 3. Identification of the ecological parameters maximizing PRLS

The maximizing function “rbga” (package “genalg” [47]), implemented in R v3.2 [48], was used to test whether at least one combination of ecological parameters lead to extensive PRLS with our model. To this end, we identified the combination of ecological parameters values able to promote the evolution of the longest PRLS, and we measured the average PRLS duration in a population which has evolved under these conditions. For each set of parameters (*α*, *β*, *δ*, *γ2* and *σ2*), a population with an initial size of 1,000 individuals was simulated and was allowed to evolve during 10,000 units of time (or 20,000 units of time, without changing the results). PRLS was the variable to be maximized in the space of parameter values. For each combination of ecological parameters, a combination of synaptic weights evolved (*i*.*e*., became the most frequent in the population). This procedure thus allowed identifying both the combination of ecological parameter values which led to the longest PRLS (referred to as EP*), and the associated synaptic weights (referred to as the best weights).

To observe and describe the allocation strategy corresponding to the best weights, a population with an initial size of 1,000 individuals was simulated using the EP*, with all individuals having the best synaptic weights, without possible mutations. With these conditions, the demographic characteristics were allowed to freely evolve during 10,000 units of time (or 20,000 units of time, without changing the results). Although individuals with the same synaptic weights necessarily have a similar strategy, decisions could vary based on the local perceived conditions. This step allowed a reduction in the inter-individual variation of realized life histories.

### 3. Testing the GMH and ECM for the emergence of menopause and extensive PRLS

To test the GMH, the same procedure was performed with the outputs corresponding to allocation in maternal care for a child who had already reproduced set to 0; grand-parenting was thus no longer a possible allocation option. Indeed, as mentioned before, grand-parenting was modeled in this study by allowing the individuals to adapt their parental investment for a given child depending on its own number of children, rather than allowing direct resource transfers to grandoffspring. If the GMH is determinant to explain the emergence of extensive PRLS, we thus expected that it cannot emerge under this condition, whatever the combination of ecological parameters. To test the ECM, the procedure was performed after removing delayed benefits of investing in cognition from the model (*i*.*e*. the integral term and the *β* parameter were removed from *Eq 3*). Indeed, without delayed benefits of investment, cognitive resources were not differentiated from somatic resources in the model (*i*.*e*. both resources are interchangeable and had the same properties).Strong delayed benefits of investment are a specificity of cognitive capital [12].Indeed, investing in neural development at time *t* promotes accumulation of skills and experience all along the life. Returns from cognitive capital can thus continue to increase (not only to be maintained) even after stopping investment in it. This is not the case for somatic capital. Indeed, although investment in somatic capital at time *t* can provide benefits later (for resource production, protection,…), these benefits will not increase without further investment. Therefore, this procedure allowed testing for the relation between cognitive resources and the duration of PRLS. Indeed, without delayed benefits of investment, cognitive resources were not differentiated from somatic resources in the model (*i*.*e*. both resources are interchangeable and had the same properties). In addition, as resource transfers are sometimes also provided by older siblings in humans, we also tested whether allowing transfers between siblings change the results.

### 4. Cost analysis

To identify the costs of suppressed or reduced PRLS in a population where extensive PRLS has already evolved, we simulated 200 populations with initial size of 1,000 individuals, where all individuals had the same allocation strategy (best synaptic weights). We allowed each population to evolve using the EP* during 20,000 units of time, without possible mutations. 100 populations were attributed to condition 1: death at age of menopause, and the 100 other populations were attributed to condition 2: one additional reproductive event at age of menopause. At *t* = 10,000, we applied the condition to half of the individuals in each population. The condition was heritable and was also applied to their offspring at each generation. No condition was applied to the other individuals and their offspring (control). For each population, the proportion of individuals who received the condition among the successive generations, up to the 10^th^, was tested for a significant departure from the expected frequency (0.5) using two-sided binomial tests (R-based function binom.test). The average fertility and the proportion of individuals who survived until reproduction were compared between the control and condition across the first ten generations using two-sided student tests. The data fitted the requirements for these tests.

## Supporting information

S1 Fig Distribution of the PRLS among the 1121 individuals who were born and died during the final 2 000 units of time of the simulation process with EP*. (PDF)
