# Supplementary figures and images for: Grandmothering and cognitive resources are required for the emergence of menopause and extensive post-reproductive lifespan
Source: PLoS Comput Biol. 2017 Jul 20;13(7):e1005631. doi: 10.1371/journal.pcbi.1005631 (PMC5519007; doi:10.1371/journal.pcbi.1005631)

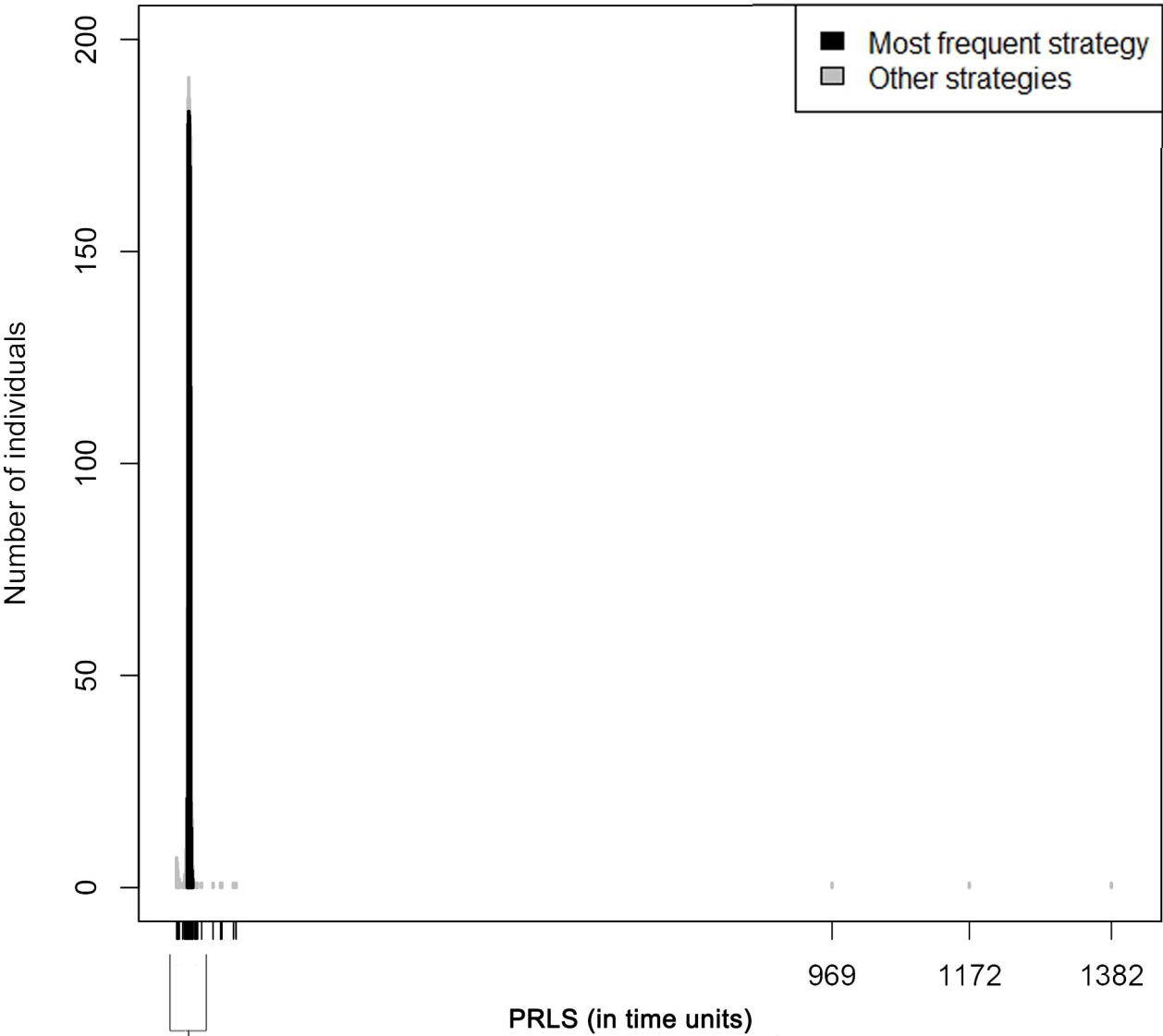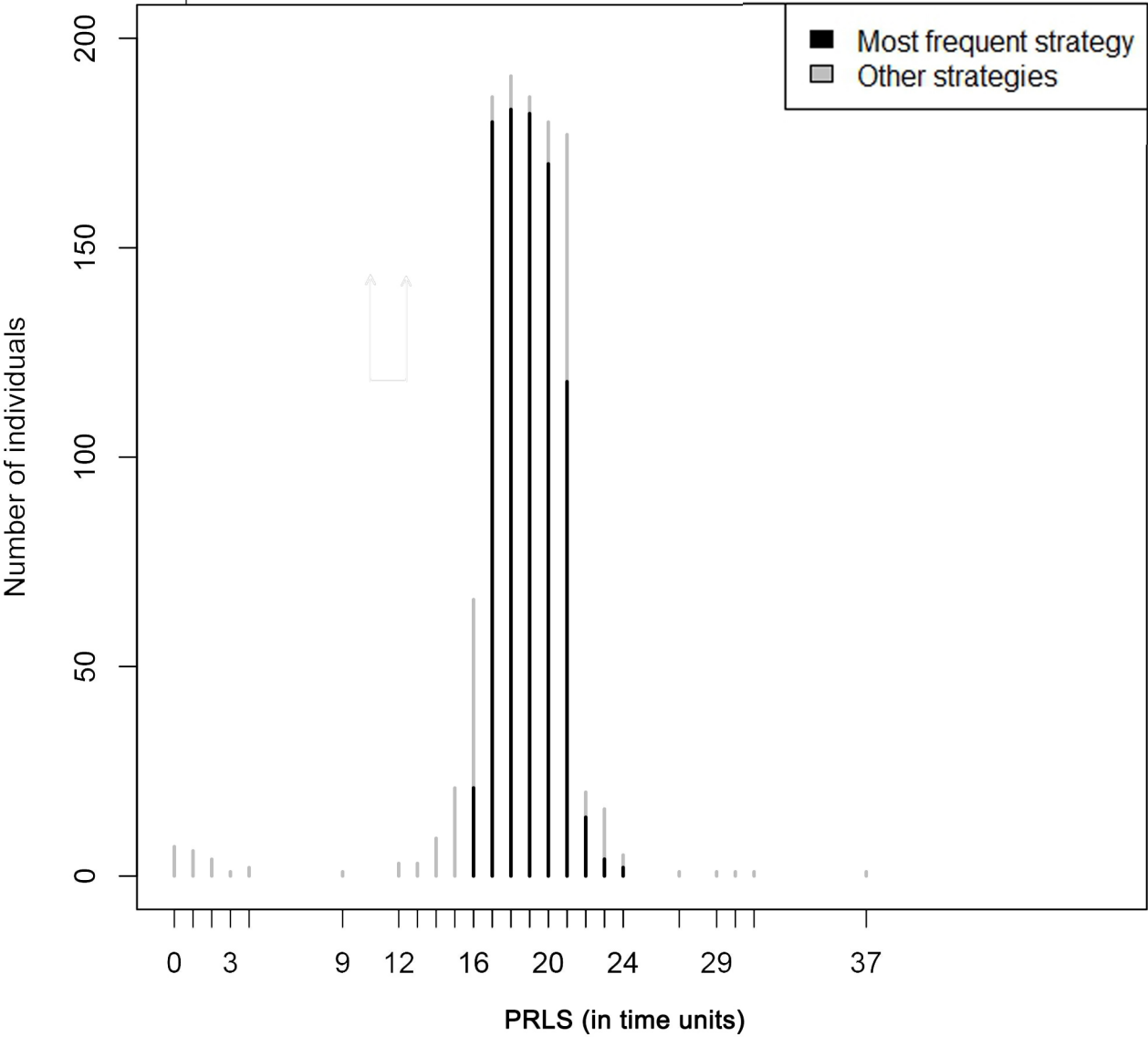

Supplement: S1 Fig — (PDF) [file pcbi.1005631.s001.pdf]
